# Accumulation of Microcystin (LR, RR and YR) in Three Freshwater Bivalves in *Microcystis aeruginosa* Bloom Using Dual Isotope Tracer

**DOI:** 10.3390/md15070226

**Published:** 2017-07-17

**Authors:** Min-Seob Kim, Yeonjung Lee, Sun-Yong Ha, Baik-Ho Kim, Soon-Jin Hwang, Jung-Taek Kwon, Jong-Woo Choi, Kyung-Hoon Shin

**Affiliations:** 1 Department of Fundamental Environment Research, Environmental Measurement & Analysis Center, National Institute of Environmental Research, Incheon 22689, Korea; candyfrog77@gmail.com (M.-S.K.); cjw111@korea.kr (J.-W.C.); 2 Marine Ecosystem and Biological Research Center, Korea Institute of Ocean Science and Technology, Ansan 15627, Korea; yeonjunglee83@gmail.com; 3 Division of Polar Ocean Environment, Korea Polar Research Institute, Incheon 21900, Korea; syha@kopri.re.kr; 4 Department of Life Sciences, Hanyang University, Seoul 04763, Korea; tigerk@hanyang.ac.kr; 5 Department of Environmental Health Science, Konkuk University, Seoul 05029, Korea; sjhwang@konkuk.ac.kr; 6 Monitoring and Analysis Division, Geum River Basin Environmental Office, Ministry of Environment, Daejeon 34142, Korea; inhtox@gmail.com; 7 Department of Marine Sciences and Convergent Technology, Hanyang University, Ansan 15588, Korea

**Keywords:** stable isotope tracer, *U. douglasiae*, *S. woodiana*, *S. arcaeformis*, toxic microcystin, *M. aeruginosa*

## Abstract

Stable isotope tracers were first applied to evaluate the *Microcystis* cell assimilation efficiency of *Sinanodonta* bivalves, since the past identification method has been limited to tracking the changes of each chl-*a*, clearity, and nutrient. The toxicity profile and accumulation of MC-LR, -RR and -YR in different organs (foot and digestive organs) from the three filter-feeders (*Sinanodonta woodiana, Sinanodonta arcaeformis,* and *Unio douglasiae*) were assessed under the condition of toxigenic cyanobacteria (*Microcystis aeruginosa*) blooms through an *in situ* pond experiment using ^13^C and ^15^N dual isotope tracers. Chl-*a* concentration in the manipulated pond was dramatically decreased after the beginning of the second day, ranging from 217.5 to 15.6 μg·L^−1^. The highest amount of MCs was incorporated into muscle and gland tissues in *U. douglasiae* during the study period, at nearly 2 or 3 times higher than in *S. woodiana* and *S. arcaeformis.* In addition, the incorporated ^13^C and ^15^N atom % in the *U. douglasiae* bivalve showed lower values than in other bivalves. The results demonstrate that *U. douglasiae* has less capacity to assimilate toxic cyanobacteria derived from diet. However, the incorporated ^13^C and ^15^N atom % of *S. arcaeformis* showed a larger feeding capacity than *U. douglasiae* and *S. woodiana*. Our results therefore also indicate that *S. arcaeformis* can eliminate the toxin more rapidly than *U. douglasiae*, having a larger detoxification capacity.

## 1. Introduction

Eutrophication in freshwater environments is a worldwide problem resulting in extensive blooms of potentially toxigenic cyanobacteria [1]. The development of cyanobacteria blooms has become a serious problem all over the world because many cyanobacteria have produced a wide variety of bioactive compounds including neurotoxins, hepatotoxins and cytotoxins in the past decades [2,3]. Among cyanotoxins, microcystins (MCs) are considered to be the most common and one of the most dangerous groups [3]. The potential toxicity of MCs [4] causes mortality and illness in both animals and humans [5] with harmful effects on many groups of aquatic organisms including zooplankton [6,7], shrimps [8], bivalves [9], and fishes [10].

The approach of top-down biomanipulation has been contrived to promote the population of zooplanktivorous fish as well as to stimulate the increase of filter feeding zooplankton, while harmful algal biomass was decreased [11,12]. The abundance of filter feeders such as mussels contributes to suppressing the formation of algal bloom and to filtering some broad range of particles, which is known as the effect of benthic–pelagic coupling that reduces planktonic biomass [13,14,15]. In addition, a wide variety of clearance rates have been reported for mussels feeding on natural seston [14,16,17].

Freshwater bivalves have a great influence on ecosystem processes where they dominate benthic biomass and coupled benthic energy and nutrient cycling [18]. By actively pumping water across their gills, they remove phytoplankton, organic matter and suspended particles from the water column, and reduce concentrations of ammonia, nitrate and phosphorous. The ecological functions performed by bivalves (e.g., filter-feeding, nutrient excretion, biodeposition, bioturbation, etc.) impact both primary producers and consumers through direct and indirect pathways. In this way, bivalves redirect energy flow and nutrient cycling in the overall lake food web [19]. Freshwater bivalves have also been extensively used in environmental risk assessment as pollutant concentrations in their tissues reflect the environmental concentrations, especially in areas with intense human activities, frequently contaminated with anthropogenic pollutant (e.g., industrial organic pollutant, sewage, manure, septic waste, fertilizer, etc.), because they live long, have a low metabolic rate, and integrate historical nutrient variation [20]. Therefore, they are good bio-indicators due to their close association with water quality, and due to time-averaged persistence of nutrient pollutants with limited movement. 

Biomanipulation with bivalves is a feasible method for management of water quality control [21,22,23]. Zebra mussels (*Dreissena polymorpha*, Pallas) have been used for a while to control algal populations [21]. North American endemic bivalve species (Unionidae) are currently under the threat of extinction because of zebra mussel attachment to their shells and food competition [24]. The use of native bivalve species in biomanipulation may therefore be preferable to the use of *Dreissena* [9]. Large freshwater bivalves (Unionidae) are reported to inhabit shallow lakes in Asian countries: China, Japan, and Korea [25]. Native bivalve organisms in Asian shallow lakes have been discovered to be widespread in European countries as well [25]. The grazing rate of unionid bivalves has been reported to have a high impact on phytoplankton densities in marine and estuarine systems since similarly-sized bivalves have been shown to decrease the phytoplankton biomass [26]. Unionids have been preferred to zebra mussels for shallow lake habitats, which mainly consist of soft substrate (mixtures of sand and mud), and often lack hard substrata. In addition, unionid mussels like *Sinanodonta* are important in nature conservation because these mussels serve as brood chambers for offspring of the bitterling, *Rhodeus sericeus* [27], which is an endangered fish species [28].

Stable C and N isotopic techniques are routinely used to examine food sources, the feeding preferences of organisms, and trophic levels [29]. Isotope labeling experiments are used to analyze ecological detail in connection with food webs. These isotope-labeling approaches could be a possible method for tracking their fates in the natural biota involving energy sources and pathways [30,31], however, use of this approach is very rare and not yet widely applied.

In the present study, the grazing impact of the three filter feeders (*Sinanodonta woodiana, Sinanodonta arcaeformis* and *Unio douglasiae*) on toxic cyanobacteria (*Microcystis aeruginosa*) was estimated from a small pond using dual stable isotope tracers. The application of ^13^C- and ^15^N-labeled phytoplankton makes it possible to directly follow the pathway and transfer of food source (cyanobacteria) into consumers (filter feeders), in contrast to past studies where only changes in compositions of chl-*a*, clearity, and nutrients were taken as the evidence for these processes. Furthermore, the distribution patterns of three common MCs (MC-LR, MC-RR, and MC-YR) concentrations in differential organs (foot and digestive gland) of the bivalves are examined to discuss the possible mechanisms underlying these patterns, with comments on the potential risk to bivalve health when these bivalves consume toxic cyanobacteria (*M. aeruginosa*).

## 2. Materials and Methods

### 2.1. Characteristics of Ponds

**Figure 1:**
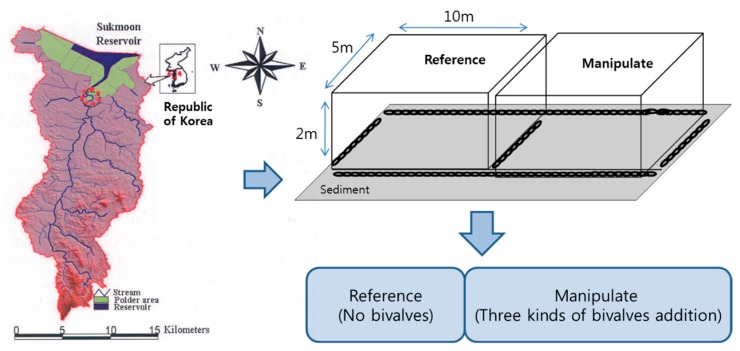
The schematic of artificial ponds in the Sukmoon wetland. The filled circle indicates the location of the study site. RP and MP had same size and capacity (2 × 5 × 10 m^3^). Three bivalves were added into MP.

To evaluate the effect of biocontrol on large cyanobacteria bloom of *M. aeruginosa* in the freshwater ecosystem, we conducted a biomanipulation test on *in situ* ponds using freshwater organisms, such as filter feeders (bivalves: *U. douglasiae, S. woodiana* and *S. arcaeformis* from the unionid family). The *in situ* pond tests were carried out from 13 October to 3 November 2008 to simulate a freshwater environment. The experimental site is located in the Sukmoon wetland (36°57′4″ N, 126°37′5″ E) in the mid-western region of South Korea (Figure 1). Ponds were classified into two types (see Figure 1): reference pond (RP) and manipulated pond (MP). Each pond enclosure was open at the surface and sealed at the bottom. They were constructed with transparent polyethylene and were suspended from frames made from PVC tubing. The enclosures were filled with reservoir water. Both ponds consisted of 100-ton tanks (10 × 5 × 2 m) and contained muddy sediment at the bottom, including reference pond (RP) and manipulated pond (MP):

(1) Reference Pond (RP; *in situ* water containing predominantly *M. aeruginosa*).
(2) Manipulated Pond with bivalves (MP; *in situ* water containing predominantly *M. aeruginosa* + bivalves (*U. douglasiae, S. woodiana* and *S. arcaeformis*)).

The filter feeders were captured from agricultural reservoirs using hand nets (mesh size <2 × 2 mm) and trawling. The mean shell length and width of the *U. douglasiae* (6.74 ± 0.51 cm and 3.82 ± 0.34 cm, respectively), *S. woodiana* (6.94 ± 0.46 cm and 3.97 ± 0.51 cm, respectively), and *S. arcaeformis* (6.78 ± 0.58 cm and 3.57 ± 0.42 cm, respectively) were slightly different; however, their standard errors overlapped. They were kept in aquaria filled with reservoir water in a layer of sand at 17~20 °C under a 16L:8D regime. Individuals were fasted for 48 h prior to the experiment. For the *in situ* pond experiment, the individual numbers of bivalves were determined through the preliminary experiments; bivalve *U. douglasiae*, *S. woodiana* and *S. arcaeformis* (5 ind. m^−2^) [32].

### 2.2. In Situ Artificial Pond Experiment

The tracer experiment was conducted when the massive toxic cyanobacteria bloom occurred in up to 89% of the phytoplankton biomass. As isotope tracers, NaHCO_3_ (Isotech; ^13^C > 99%) and (NH_4_)_2_SO_4_ (Isotech; ^15^N > 99%) were added to each pond. The ^13^C content of the dissolved inorganic carbon (DIC) pool was increased up to about 5% and the ^15^N content of the dissolved inorganic nitrogen (DIN) pool was also stabilized by enriching with at most 10% every 48 h. Next, filter feeders (*U. douglasiae, S. woodiana* and *S. arcaeformis*) were added into the MP. The samples of water and bivalves were collected at the 1st, 2nd, 3rd, 4th, 5th, 6th, 8th, 10th, 13th, 16th and 21st days after the addition.

### 2.3. Analysis of Water Quality Parameters

Two replicates of water samples were collected to determine the concentrations of dissolved inorganic nitrogen (DIN: NH_4_^+^, NO_3_^−^, NO_2_^−^) and dissolved inorganic phosphate (DIP: PO_4_^3−^) in each pond. The concentrations of DIN and DIP were measured by standard colorimetric techniques following the methods of Strickland and Parsons [33] using a UV-spectrophotometer (Varian Carry 50, Houston, TX, USA). The Chl-*a* concentration was determined by using a fluorescence spectrophotometer (Turner Design, 10R, San Jose, CA, USA) after extraction with 90% acetone for 24 h. Water temperature was measured using a multi-parameter water quality sensor (YSI Environmental Monitoring System 660, Yellow Springs, OH, USA). All environmental parameters including nutrients, chl-*a*, and water temperature were monitored during the experimental period.

### 2.4. Enumeration and Biomass Determination of Phytoplankton

Water samples for the enumeration of phytoplankton cells were sedimented for 24 h, and a known volume of the concentrated sample was placed in a Sedgewick–Rafter counting chamber, where at least 300 cells were counted under ×200–400 magnification. Taxa (for example, *Microcystis* sp.) were enumerated only as larger units. Phytoplankton cell density was then determined on the basis of geometric solids that closely approximated each cell or colony shape [34].

### 2.5. Analysis of Stable Isotope Ratios

To analyze the stable isotope ratios of particulate organic matter (POM; mostly phytoplankton), water samples were passed through 20 μm mesh to remove zooplankton, and the remaining water was filtered through pre-combusted (450 °C, 24 h) glass fiber filters (Whatman GF/F, Sigma-Aldrach, Yong In, Korea) using gentle vacuum filtration. The filter samples were fumed for 24 h with saturated HCl to remove inorganic carbon and were dried completely using a freeze drier. The bivalve sampling was carried out using hand nets (mesh size: 2 × 2 mm) in the small pond. They were dissected in order to separate the digestive gland, stomach, muscle (abductor), gill, and mantle (three samples for each tissue). All tissue samples were freeze-dried and then ground into a fine powder using a grinder (FRITSCH-planetary mono mill, Pulverisette 6, Idar-Oberstein, Germany). The freezing and storage processes do not affect δ^13^C and δ^15^N values of biota tissue [35]. Homogenized powder samples of each tissue were decalcified with 1N HCl for at least 24 h to remove possible carbonates. However, subsamples for δ^15^N analysis were not treated with acid because it has been reported that HCl treatment affects δ^15^N values [36]. After the acid treatment, the samples were re-dried using a freeze drier and were ground to a fine powder, which was thoroughly mixed prior to analysis. Measurements of stable carbon and nitrogen isotopic ratios were performed with a continuous flow isotope ratio mass spectrometer (Isoprime; GV Instrument, Manchester, UK) coupled with an elemental analyzer (Euro EA 3000-D, Milan, Italy). Isotopic ratios were presented as δ values (‰) related to the Vienna PeeDee Belemnite (VPDB) standard and to atmospheric N_2_ for carbon and nitrogen, respectively. The reference materials were IAEA-CH6 (δ^13^C = −10.45 ± 0.04‰) and IAEA-N1 (δ^15^N = 0.4 ± 0.2‰). The analytical precision was within 0.2‰ and 0.3‰ for carbon and nitrogen, respectively. Isotope ratios were reported in per mil (‰) using standard delta notation, using Equation (1):

δ X = [(R_sample_ − R_std_)/R_std_] × 1000(‰) (1)

where X = ^13^C or ^15^N, R = ^13^C/^12^C or ^15^N/^14^N, and std (standard) = Vienna Peedee Belemnite (VPDB) for carbon or air N_2_ for nitrogen. The δ-values were converted to atom %, which is more appropriate for labeled samples. Conversion was performed according to Equation (2):

A (atom %) = 100/{1/[(δ sample/1000 + 1) × A_ns_] + 1} (2)

where the A_ns_ for carbon is 1.118 × 10^−2^ and that for nitrogen is 3.677 × 10^−3^.

### 2.6. Determination of MCs Concentration and Production Rate

A bivalve sample was screened for the presence of neurotoxins and hepatotoxins, but since these were never found only results on MCs will be presented here. We choose the three most common MC compounds (-LR, -RR, -YR); this is also the case in the great majority of studies dealing with the presence and effects of toxicity on many aquatic organisms, thus enabling comparison with other results. The purification and analysis of MCs were carried out using the methods developed by Harada et al. [37]. From each sample of freeze-dried *Microcystis* cell material and filter feeders tissue, the MCs were extracted twice with 20 mL of 5% (*v*/*v*) acetic acid for 1 h while shaking at 140 rpm. The extract was centrifuged at 12,000× *g*, and then the supernatant was applied to a C18 cartridge (Sep-Pak; Waters Association, Milford, MA, USA). The cartridge was rinsed with water and 20% methanol in water. The eluate from the cartridge with 90% methanol in water was evaporated to dryness, and the residue was dissolved in 100% methanol. The sample solution was analyzed on an HPLC (Agilent Technologies 1200 series, Santa Clara, CA, USA). The separation was performed on an ODS column (Cosmosil 5C18-AR, 4.6 mm × 150 mm) reverse-phase column and the mobile phase was a 0.1% formic acid:water solution with constant flow at 1 mL min^−1^. The MCs were identified by their UV spectra and retention times, and by spiking the sample with purified standards of MC-LR (Sigma, Sigma-Aldrach, Yong In, Korea), MC-YR (Sigma, Sigma-Aldrach, Yong In, Korea) and MC-RR (Sigma, Sigma-Aldrach, Yong In, Korea). MCs concentrations were determined by comparing the peak areas of the test samples with those of the standards available (MC-LR, MC-YR and MC-RR, Sigma, Sigma-Aldrach, Yong In, Korea).

### 2.7. Statistical Analysis

All data were tested for normality and homogeneity of variances (Levene’s median test). A paired-difference *t* test was used to analyze the relationship of environmental factors between two ponds. A *p*-value of less than 0.05 was considered as statistically significant. All statistics were performed using SPSS software (version 22.0, SPSS Inc., New York, NY, USA).

## 3. Results

### 3.1. Water Quality Condition in an Artificial Enclosed Pond

**Figure 2:**
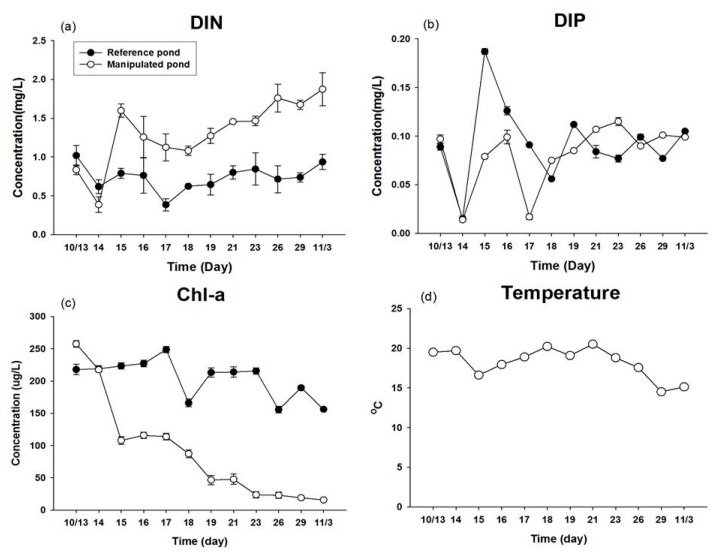
Mean concentration values of (**a**) DIN; (**b**) DIP; (**c**) Chl-*a*; and (**d**) temperature in RP and MP.

Biocontrol with bivalves ponds (MP) resulted in significant differences in water quality parameters such as Chl-*a* (*p* < 0.001) and DIN (*p* < 0.001) concentration in the cyanobacteria blooming environment in comparison with those of the RP pond (Table 1). The DIN concentration of the MP by the third day was maintained at the range of 1.5 and 1.8 mg L^−1^, which was higher than the RP since the phytoplankton exhausted the nutrients from the water column through photosynthetic activity in RP, while the filter feeders could release nutrients as fecal pellets into the water column in MP (Figure 2a). The DIP concentration of the RP was higher until the fifth day, but fluctuation was observed from the sixth day to the end of the experimental period, probably due to resuspension of the phosphorus from sediment through windy turbulence and organism movement (Figure 2b). The Chl-*a* concentration in MP was dramatically decreased compared with that of the reference pond after the beginning of the second day, with a range of 15.6–217.5 μg L^−1^ (Figure 2c). This indicates that phytoplankton containing toxic cyanobacteria (*M. aerugionsa* and *A. spiroides*) were eliminated by filter feeders as their prey. The water temperature showed the same variation in both ponds, ranging from 16 to 24 °C (Figure 2d).

### 3.2. The Composition and Biomass Variation of Phytoplankton

**Figure 3:**
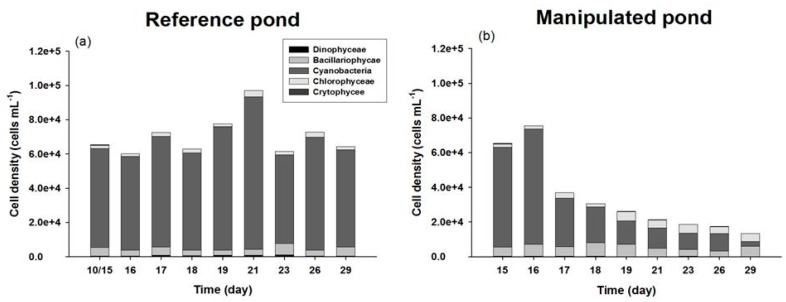
Temporal variation of phytoplankton groups to each total biomass in the two different ponds: (**a**) RP and (**b**) MP.

Biocontrol with bivalves ponds (MP) resulted in significant differences in phytoplankton cell density (*p* < 0.005) in the cyanobacteria blooming environment in comparison with those of the RP pond (Table 1). The relative proportions of phytoplankton species at the beginning of experiment showed that most dominant species were cyanobacteria (87.8%) and bacillariophycae (7.9%), followed by crytophycee (0.7%), chlorophyceae (3.0%) and dinophyceae (0.5%) (Table 1). Species of cyanobacteria were composed of *M. aerugionsa, A. spiroides, Synechocystis pevalekii, Aphanocapsa elachista,* and *Chroococcus* sp. in both ponds at the beginning of the experiment (Table 2). The cell density of phytoplankton in the RP maintained a constant biomass with small fluctuations during the entire experiment, but dramatically decreased in the MP (Figure 3). At the end of the experiment, the relative proportion of cyanobacteria species in the RP showed a 6% difference, from 88% to 82%, but the MP had a 67% difference, from 88% to 21% (Figure 3). These results supported the biomanipulation effect of multiple filter feeders in reducing cyanophyceae biomass.

### 3.3. Measurement of ^13^C and ^15^N Atom % of Filter Feeders

**Figure 4:**
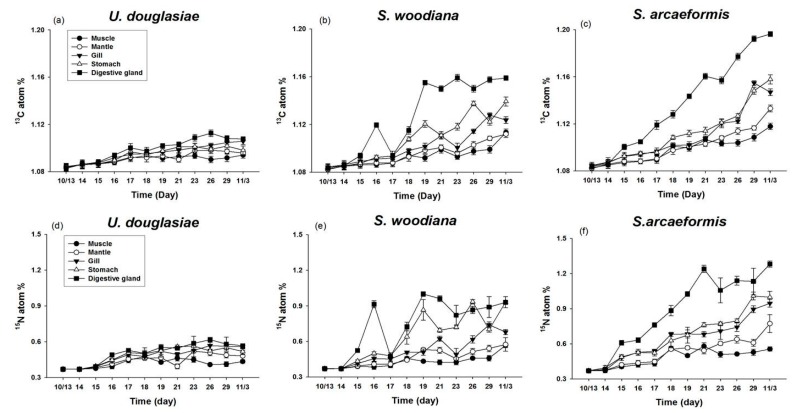
Incorporation of ^13^C atom (%) in (**a**) *U. douglasiae*, (**b**) *S. woodiana*, and (**c**) *S. arcaeformis* and ^15^N atom (%) in (**d**) *U. douglasiae*, (**e**) *S. woodiana*, and (**f**) *S. arcaeformis* tissues in the RP during the study period.

**Figure 5:**
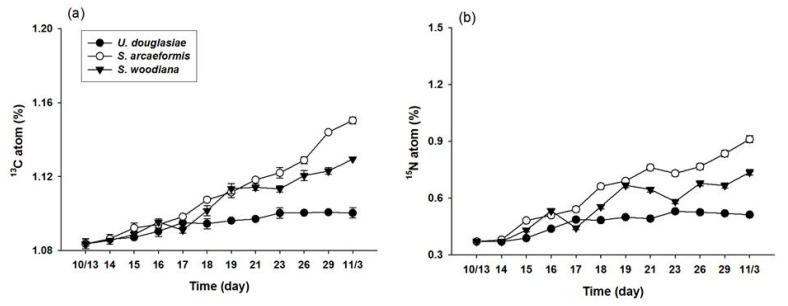
Incorporation of (**a**) ^13^C and (**b**) ^15^N atom (%) in each bivalve (*S. woodiana, S. arcaeformis* and *U. douglasiae*) in the MP during the study period.

Most filter feeders showed continuous apparent enrichment of ^13^C and ^15^N tracers in their tissues during the experimental period (Figure 4). Distinguished by filter feeder species, in the MP, *U. douglasiae* showed 1.08~1.11 ^13^C and 0.36~0.61 ^15^N atom % respectively, *S. woodiana* showed a 1.08~1.15 ^13^C and 0.36~0.99 ^15^N atom % respectively, and *S. arcaeformis* showed a 1.08~1.20 ^13^C and 0.36~1.28 ^15^N atom % respectively, demonstrating that *S. arcaeformis* has higher assimilation efficiency for toxic cyanobacteria cells through feeding activity (Figure 5). The different atom percentages might indicate different assimilation capacities among the freshwater filter feeder species. The filter feeders showed different isotopic enrichment among the tissues, showing more enrichment in the digestive gland and stomach but relatively low incorporation rates in mantle and gill, indicating that each organ had differential turnover rates (Figure 4).

### 3.4. MC Concentration of POM and Three Kinds of Bivalves

**Figure 6:**
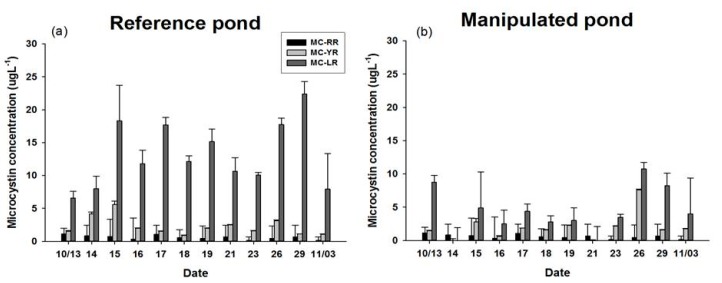
MCs (MC-RR, -YR, and -LR) concentrations of POM in (**a**) RP; (**b**) MP.

The MC concentrations of the POM in the RP and MP showed remarkable variation (Figure 6). Within two days of beginning the experiment, the MC concentration showed a remarkable decrease of the POM in the MP while the RP maintained a relatively constant MC concentration through the end of the experiment. However, those values did increase slightly, possibly due to release from filter feeders as fecal pellets or expelled cyanobacteria cells in water column at last 7 days.

**Figure 7:**
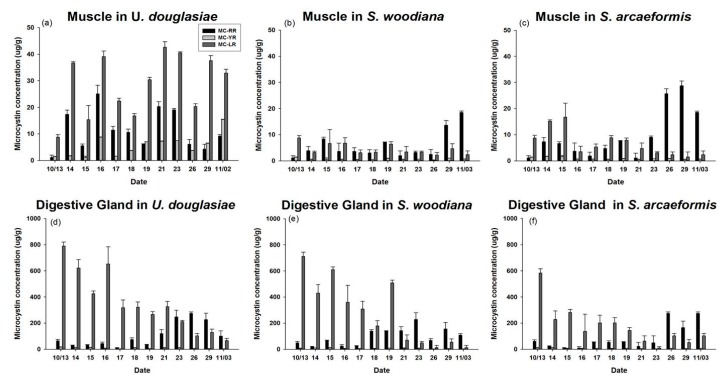
MCs (LR, RR and YR) concentrations in bivalve muscle tissue: (**a**) *U. douglasiae*, (**b**) *S. woodiana*, and (**c**) *S. arcaeformis*; and digestive gland tissue: (**d**) *U. douglasiae*, (**e**) *S. woodiana*, and (**f**) *S. arcaeformis*, in the MP during the study period.

Predation by *U. douglasiae*, *S. woodiana,* and *S. arcaeformis* on the toxic cyanobacteria resulted in a clear increase of the MC concentration in their body tissues (Figure 7). The total MC concentration of *U. douglasiae* showed higher values of 11.2~70.1 μg·g^−1^DW (Dry Weight) in muscle tissue and 168.9~869.0 ng·g^−1^DW in gland tissue, while *S. woodiana* and *S. arcaeformis* showed lower values of 6.3~30.8 ng·g^−1^DW in muscle and 83.4~766.5 ng·g^−1^DW in gland, and 5.9~21.5 ng·g^−1^DW in muscle and 61.2~654.8 ng·g^−1^DW in gland, respectively. The highest amount of MC was incorporated into muscle and gland tissues in *U. douglasiae* during the study period, nearly two or three times higher than shown in *S. woodiana* and *S. arcaeformis.* Also, the MC concentration in gland tissue among the filter feeders was substantially higher compared to that in muscle tissue, due to differential turnover rates.

## 4. Discussion

### 4.1. Dynamics of Feeding Activity and MCs Concentration in Filter Feeders

In this study, *t* tests of environmental variables means such as DIN (*p* < 0.001), Chl-*a* (*p* < 0.001) concentration and phytoplankton cell density (*p* < 0.005) showed that there were significant differences between the RP and MP ponds (Table 1). Chl-*a* and DIN concentration were less in both MP ponds compared with those in the RP pond. This suggests that three kinds of bivalves preyed on cyanobacteria (*M. aeruginosa*), which resulted in greater water clarity. However, reduced Chl-*a* concentration may not be direct evidence of bivalves assimilation of *M. aeruginosa* because bivalves may expel toxic cyanobacteria without digesting them in the form of feces or pseudofeces that is released into the water column or sinks to the bottom. Therefore, the grazing efficiency of bivalves was evaluated according to the ^13^C and ^15^N atom % incorporated into the biota.

This study is the first to directly compare the assimilation rates and MC concentrations of three different kinds of filter feeders in *M. aerugionsa* blooms. The inorganic forms of ^13^C and ^15^N tracers were assimilated into the cyanobacteria body as organic forms through photosynthesis, and synthesized new phytoplankton cells. The atom % increase of filter feeders indicates that newly synthesized phytoplankton cells were assimilated into the organism’s cells through feeding activity. The ^13^C and ^15^N atom % of three kinds of filter feeders showed continuous apparent enrichment (Figure 4) and apparent increases of MC concentration in their body tissues (Figure 7). Also, the Chl-*a* concentration and phytoplankton biomass in the MP decreased throughout the study period (Figure 2 and Figure 3). These results indicate that filter feeders have a large capacity to assimilate a cyanobacteria-derived diet. Filter feeders such as bivalves can filter a broad size range of particle diameters, significantly reduce phytoplankton populations, and greatly increase water clarity [14]. In particular, unionid bivalves can filter large amounts of the water column and decrease phytoplankton densities in cases of large biomass [26,38]. Therefore, active filtration was exhibited by unionid filter feeders in this study, and led to declines in phytoplankton biomass, even in a large water body.

However, filter feeders cannot avoid the accumulation of toxins in their body from ingestion of toxic cyanobacteria during a bloom period. The distinction between ^13^C and ^15^N atom % among the filter feeders shows contrasting ingestion rates due to differential incorporation of *Microcystis* cells in their body tissues. To elucidate the different ^13^C and ^15^N atom % and MC concentrations among the three filter feeders (*U. douglasiae, S. woodiana* and *S. arcaeformis* for the family of unionid) (Figure 4 and Figure 7), toxic MCs bioaccumulation and its depuration rate need to be considered because toxic *Microcystis* impacts the prey ingestion rate of bivalves [9]. Yokoyama and Park [39] have reported that the concentration of bioaccumulated MC in *S. woodiana* bivalves was very low compared to that in *U. douglasiae* bivalves, claiming that *S. woodiana* might reject toxic *Microcystis* as pseudofeces prior to ingestion. However, in our study, *U. douglasia* bivalves might expel more toxic *Microcystis*, because ^13^C and ^15^N atom % values in *S. woodiana* and *S. arcaeformis* were higher in comparison with those of *U. douglasiae* bivalves. These results suggest that *S. woodiana* and *S. arcaeformis* would continuously ingest newly photosynthsized *Microcystis* cells into their body, but they may have an immunological system for depuration of toxic MCs, unlike *U. douglasiae. U. douglasiae* assimilated *Microcystis* cells, but those were expelling bioaccumulated *Microcystis* cells into the water column, suggesting its limited ability to incorporate *Microcystis* cells (Figure 5).

In the present study, we observed a rapid uptake of MC by filter feeders, since toxins were detected in muscle and gland tissues shortly after the beginning of the cyanobacteria bloom (Figure 7). Direct toxin transference to the filter feeders through oral ingestion can be interpreted from the existence of cyanobacteria in the water column. Among the filter feeders, the highest amount of MC was found in *U. douglasiae*, nearly two or three times higher than shown in *S. woodiana* and *S. arcaeformis* (Figure 7)*,* reporting studies of MC bioaccumulation in bivalves implemented in both laboratory [40,41,42] and field [14,39,43]. Similar results have been found in other studies: Yokoyama and Park (2003) reported extremely high accumulations of MC-LR in *U. douglasiae* through laboratory experiment. Chen et al. [44] reported that toxic cyanobacteria application to *S. woodiana* resulted in lower accumulated hepatopancreas concentrations of MC, in comparison with three other kinds of filter feeders. Watanabe et al. [43] reported that *S. woodiana*, *U. douglasiae* and *Cristaria plicata* accumulated *Microcystis* at a range of 1 ± 5 mg/mussel during a large *Microcystis* bloom. Hepatopancreas tissues for *S. woodiana* showed lower MC-LR values (0.21 μg·g^−1^DW) in Lake Suwa in Japan, compared to MC-LR values (2.7 μg·g^−1^DW) in *U. douglasiae*. Moreover, Yokoyama et al. [39] reported that MC was detected at much higher concentrations in *U. douglasiae* (420 μg·g^−1^DW) than in *S. woodiana* (12.6 μg·g^−1^DW).

We found similar tendencies to past studies: it is clear that MC were detected at much higher concentrations in *U. douglasiae,* compared to in *S. woodiana* and *S. arcaeformis* (Figure 7). MC bioaccumulation in *U. douglasiae* was influenced not only by intracellular MC in toxic cyanobacteria (*M. aeruginosa*) but also by their depuration rate. These different bioaccumulation patterns among the filter feeders can be interpreted as interspecific differences in selective ingestion, MC metabolism, and depuration rate. First, selective ingestion by filter-feeding bivalves is a well-known feeding mechanism. The bivalves were capable of filtering a wide size range of sestonic particles. Ingestible particles are directed straight into the mouth, whereas unwanted particles are sorted on the gills and palps, then expelled as pseudofeces in the surrounding water [17]. However, we expect that these results were caused not only by selective grazing but also by the prey density effect, because of the ~90% composition of toxic cyanobacteria in the total phytoplankton abundance. Based on our study, unionid families could not ingest the toxic cyanobacteria as a prey even though they could selectively ingest prey, because of the impact on their physiological condition by harmful compounds (MCs).

Second, based on our results, it is clear that *S. woodiana* and *S. arcaeformis* bivalves differ from *U. douglasiae* in MC metabolism, which is involved with uptake and depuration. Possibly *U. douglasiae* depurated toxins more slowly over the same time, since its MC concentration was higher than in *S. woodiana* and *S. arcaeformis*. This differs from our results, which suggest MC may be more resistant to degradation in the *U. douglasiae,* as suggested by higher MC concentration in both organs. This kind of depuration process may be a result of cycles in the production and degradation of a protein phosphatase–MC adduct [45]. The activity of detoxification enzymes such as glutathione-*S*transferase and glutathione peroxidase has been shown to increase in embryos of zebra fish (*Danio rerio*) exposed to MC-LR [46]. Pflugmacher et al. [47] demonstrated that MC was detoxified as a result of conjugation with glutathione in various aquatic organisms including zebra mussels, fish, and daphnid. Therefore, our results demonstrated that *S. woodiana* and *S. arcaeformis* would ingest toxic cyanobacteria, but they may have an immunological system that allows them to depurate toxic MCs in contrast to *U. douglasiae*. Also, MC-LR concentration was lower in muscle tissue in both *S. woodiana* and *S. arcaeformis* bivalves during the 14 days from the beginning of the experiment, while those values increased after 14 days. The increased MC-LR concentration might indicate that the detoxification activity of MC-LR decreased, leading to excretion or physiological degradation. It is known that MC-LR can conjugate with glutathione (GSH) or cysteine and ultimately degrades to MC-LR-Cys, consequently reducing the toxicity and enhancing the excretion of MC-LR [48]. Therefore, conjugation of MC-LR with cysteine or GSH in muscle tissue might be an important pathway for the detoxification and excretion of MC-LR in *S. woodiana* and *S. arcaeformis*. For future study, to better understand the role of MC-GSH and MC-Cys conjugates in the detoxification of MCs in bivalves, quantitative evaluation of such conjugates in filter feeder tissues is needed.

### 4.2. Stable Isotope Fractionation in Differential Organs

The filter feeders showed different isotopic enrichment among their tissues, showing more enrichement in their digestive glands and stomach organs but relatively low incorporation rates in mantle and gill (Figure 4). A distinction of isotopic ratios and turnover rates for each tissue has been observed in mussels *Pecten masimus* [49] and *Mytilus galloprovincialis* [50]. Incorporation reflects the metabolic breakdown of old tissue and subsequent replacement by tissue synthesized from the new diet. Raikow and Hamilton [51] demonstrated that stable isotope enrichment of the tissues (gland and muscle) of unionid bivalves reflected short-term and long-term assimilation of nutrients. Different isotopic enrichment of tissues from three bivalves showed more enriched atom % in the digestive glands and stomach organs during the entire experimental period because those organs have relatively faster turnover rates and reflect short-term assimilation records [51,52] (Figure 4). The muscle organs of the bivalves showed lower enriched atom % because of their slow turnover and relatively long-term integration of energy source [51]. Mantle and gills tissues also indicated relatively low incorporation rates, because these organs have slow turnover rates, integrating diet isotopic signatures over a longer time period than other tissues, such as digestive glands, that have faster turnover rates [4,49]. Hill et al. [53] reported that mantle tissue replaced its carbon isotopes faster than gill parts, while gill tissue was faster than adductor muscle in the mussel *Perna perna*, because mantle is the main energy source for sustaining gonadal development. However, a differential tendency of a faster turnover rate was observed in gills compared to mantle organs (Figure 4). Mantle organs in unionoid bivalves in Korean lakes and reservoirs metabolically synthesized a little active gonad, due to the warm season for spawning from April to June. Therefore, incorporated ^13^C and ^15^N atom % in mantle organ showed lower values than in gill organs, since our studied species rarely breed during the study period.

### 4.3. Management and Implications for Water Quality

Among the freshwater bivalves, zebra mussels (*D. polymorpha*) showed higher clearance rates in single cells of the *Microcystis* [54], with a reverse gradient between mussel densities and the cyanobacteria biomass [55]. Therefore, mussels could be used as a potential tool in the biomanipulation of shallow lakes suffering from harmful cyanobacteria blooms [21,56]. However, Vanderploeg et al. [14] have argued that *D. polymorpha* promoted the return of *Microcystis* blooms, because they selectively ejected colonies of *Microcystis*. These mussels make a seston selection but respond differently to single cells or colonies of cyanobacteria. Non-native mussel species (*D. polymorpha*) are a threat to native mussels because invasive species often differ greatly from native species in resource use and trophic interactions; they have great potential to negatively affect ecosystems [57,58]. Therefore, unionids are better adapted to shallow lakes or reservoirs with soft substrates than zebra mussels, which prefer hard substrate for settlement. Native bivalve species (unionids) are recommended instead of invasive species (*D. polymorpha*) for biomanipulation on harmful cyanobacteria blooms in lakes or reservoirs. Unionid mussels can filter large amounts of the water column, as long as their biomass is large [38]. However, the research on common native freshwater bivalves (unionid mussels, like *Sinanodonta* sp. and *Unio* sp.), especially under toxic cyanobacteria bloom conditions, is still lacking. Therefore, this study provided the responses of bivalve species in mesocosm to evaluate their bio-control effects. Xie et al. [59] and Yokoyama [39] have revealed that *Sinanodonta* bivalves persist intoxic cyanobacteria bloom and show lower bioaccumulation patterns of MC. *Sinanodonta*’s grazing capacity per individual is equal to or higher than that of *D. polymorpha* [9]. For example, *Sinanodonta* bivalves showed × 12 higher efficiencies of clear unit per square meter per unit of water volume than *Dreissena* mussels [9,17]. *Sinanodonta* bivalves had a larger effect on the primary productivity of toxic cyanobacteria, demonstrating a more realistic achievement for biomanipulation. Also, *Sinanodonta* (*S. arcaefomis* and *S. woodiana*) should incorporate more toxic cyanobacteria cells than *U. douglasiae*, probably due to its larger detoxification capacity. As a result, *S. arcaefomis* and *S. woodiana* could be useful organisms to reduce massive toxic cyanobacteria blooms in eutrophic agricultural reservoirs and lakes. This is the first study using stable isotope tracers to evaluate the *Microcystis* cell assimilation efficiency of *Sinanodonta* bivalves. 

## 5. Conclusions

We conclude from our study that *S. arcaefomis* is the most efficient grazer of toxic cyanobacteria. *S. arcaeformis* can eliminate the toxin more rapidly than *U. douglasiae*, probably due to its larger detoxification capacity. However, *U. douglasiae* has a smaller capacity to assimilate toxic cyanobacteria derived from diet, and is affected by the occurrence of toxin-producing cyanobacteria bloom. For biomanipulation purposes, we suggest that the use of *Sinanodonta* deserves more attention since this is a native (endemic) bivalve, contrary to *U. douglasiae*, which probably is not suitable for biomanipulation that involves the removal of toxic cyanobacteria.

## Figures and Tables

**Table 1:** Statistical *t* test results for environmental parameters between the RP and MP pond (*n* = 5), (* *p* < 0.05). RP: Reference pond, MP: Manipulated pond.

Parameters (Unit)	*p* Value
Water temperature (°C)	0.962
Dissolved inorganic nitrogen (mg L^−1^)	0.000 *
Dissolved inorganic phosphorus (mg L^−1^)	0.364
Chl-*a* (μg L^−1^)	0.000 *
Phytoplankton cell density (cells mL^−1^)	0.005 *

**Table 2:** Major species and relative proportions (%) of phytoplankton taxa in the initial pond in the Sukmoon wetland at the beginning of the experiment.

Class	Species	Cell Density (Cells mL^−1^)	Relative Proportion (%)
Cyanobacteria			87.84
	*Microcystis aeruginosa*	43,600	
	*Anabaena spiroides*	8560	
	*Synechocysits pevalekii*	2240	
	*Aphanocapsa elachista*	1548	
	*Chroococcus* sp.	1600	2.99
Chlorophyceae			
	*Scenedesmus* sp.	1280	
	*Pediastrum boryanum*	0	
	*Pediastrum simplex*	0	0.73
	*Staurastrum* sp.	320	0.54
	*Monoraphidium contortum*	360	
Crytophyceae			
	*Cryptomonas erosa*	480	7.87
Dinophyceae			
	*Ceratium hirundinella*	360	
	*Euglena* sp.	0	
Bacillariophyceae			
	*Synedra acus*	440	
	*Synedra* sp.	160	
	*Navicula* sp.	200	
	*Aulacoseira granulata*	2280	
	*Aulacoseira* sp.	240	
	*Nitzschia holsatica*	400	
	*Cyclotella comta*	1440	
